# Letter to the Editor: On the term ‘interaction’ and related phrases in the literature on Random Forests

**DOI:** 10.1093/bib/bbu012

**Published:** 2014-04-09

**Authors:** Anne-Laure Boulesteix, Silke Janitza, Alexander Hapfelmeier, Kristel Van Steen, Carolin Strobl

**Keywords:** random forest, statistics, interaction, correlation, conditional inference trees, conditional variable importance

## Abstract

In an interesting and quite exhaustive review on Random Forests (RF) methodology in bioinformatics Touw *et al.* address—among other topics—the problem of the detection of interactions between variables based on RF methodology. We feel that some important statistical concepts, such as ‘interaction’, ‘conditional dependence’ or ‘correlation’, are sometimes employed inconsistently in the bioinformatics literature in general and in the literature on RF in particular. In this letter to the Editor, we aim to clarify some of the central statistical concepts and point out some confusing interpretations concerning RF given by Touw *et al.* and other authors.

## INTRODUCTION

Random Forests (RF) is a valuable analysis tool, especially in situations where datasets contain many variables with complex relationships. Therefore, many authors use statistical terms such as ‘interactions’ and ‘conditional relationships’ to indicate the complexity of the data—yet without clearly defining their meanings or, alternatively, they use these terms inconsistently throughout their paper [1–3]. Here, we will give a consistent statistical definition of those concepts that are most central for understanding the rationale and behavior of RF. In examining these definitions, we explain some of the statements of Touw *et al.* [1] that we found unclear when reading their paper. We stress, however, that some terms can have sensible meanings other than those outlined in our letter. Our intention is not to impose our definitions on everyone but rather to provide a possible interpretation of the considered concepts that allows a better understanding of some aspects of RF methodology. We aim to point out that it is important to define concepts clearly and consistently within every article, no matter whether formal statistical terms are used for that purpose or not.

## INTERACTION, CORRELATION AND CONDITIONAL DEPENDENCE: WHAT DO THEY MEAN?

### Interactions and effect modification

The term ‘interaction’ is related to the concept of (effect) modification. According to Miettinen [4], effect modification is present when the measure of association between a predictor variable [e.g. a single nucleotide polymorphism (SNP)] and the response variable (e.g. a trait) is not constant across another characteristic (e.g. population strata or a SNP at a second locus). Because such a characteristic changes the effect of the SNP of interest on the trait, this phenomenon is often referred to in the literature as effect modification. Notably, some epidemiology textbooks [5] reserve the term effect modification for when the modification is linked to a causal mechanism and use the reduced term modification otherwise. There seems to be no consensus on how to define the term ‘effect modification’ in the literature [6–8].

The statistical literature avoids these definition problems by using the term ‘interaction’ in the context of regression models with additive effects to denote deviations from the additive model that are reflected by the inclusion of the product of at least two predictor variables in the model. In this letter we take this perspective, which has the advantage that it involves unambiguous definitions; see the next section for a formal definition of interaction effects within a regression model.

In many of the explanations below it is important to clearly distinguish between the response variable *Y* of a supervised learning problem, also termed outcome, criterion variable or dependent variable and the potential predictor variables 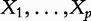, also called features, covariates or independent variables. Observed values of *Y* and *X_j_* are denoted by *y* and *x_j_*, 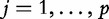, with *n* denoting the number of observations and *p* the number of predictor variables.

### Interactions in regression models

Let us consider a regression problem with a response variable *Y* and two predictor variables *X*_1_ and *X*_2_. If *Y* is binary, a popular statistical approach to build a prediction model for *Y* based on *X*_1_ and *X*_2_ is the logistic regression model, which relates the probability 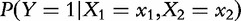 to a linear combination of the predictor variables through the so-called ‘logit’ function 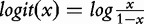. In this context, interaction effects are modeled by including a separate effect 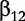 for the interaction in the linear combination

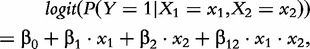

where 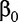, 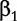, 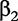 and 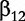 denote parameters that link the predictors *X*_1_ and *X*_2_ to the response variable *Y* and have to be estimated from the data at hand. It is clear from this formula that, if the parameter 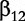 is non-zero, the effect of *x*_1_ on 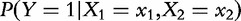 depends on *x*_2_, because the linear combination can be reformulated as

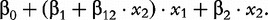

Likewise, the effect of *x*_2_ depends on *x*_1_. Looking at this formula, it is intuitive that the notion of interaction is equivalent to one of the possible definitions of the notion of effect modification: the value of *x*_1_ modifies the effect of *x*_2_ and vice versa.

Note that this classical statistical way of viewing interactions is in line with Fisher’s original 1918 definition of epistasis (alias: genetic interaction, [9]), which involves a statistical interaction between two variables *X*_1_ and *X*_2_, each coding allelic information at a different genetic locus.

A simplified example of such an interaction could be the probability of correctly assessing fetal health during pregnancy (response variable *Y* with *Y *= 1 for correct diagnosis, *Y* = 0 for incorrect diagnosis). A correct assessment is possible only if high-quality ultrasound devices are available (predictor variable *X*_1_) and if the hospital staff is trained to use them and interpret the pictures (predictor variable *X*_2_). This is an interaction effect, because only both predictor variables together can explain whether the fetal health can be assessed correctly: intuitively, the coefficient 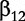 of the product 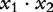 will be high, because it is important that both *x*_1_ and *x*_2_ equal 1 for the diagnosis to be correct. Only if the staff is adequately trained on their use does the availability of high-quality ultrasound devices have an effect on the correct assessment of fetal health and vice versa. In addition to the interaction effect, it is plausible in this example that the variable ‘trained staff’ also has a main effect on the correct assessment of fetal health, because a well-trained physician might partly assess fetal health using other methods (e.g. listen to the fetal heartbeat even in the absence of an ultrasound device). Conversely, the availability of ultrasound devices has no main effect, because the presence of trained staff is absolutely necessary for an ultrasound device to be useful.

### Interaction and association/correlation

It is important to note that an interaction is not the same as ‘confounding’. We know from our experience in statistical consulting for applied scientists without strong quantitative background that the concepts are sometimes confused.

Confounding may occur when a variable is associated with both the predictor variable of interest and the dependent variable. Note that the term ‘correlation’ (referring to Pearson’s correlation) is often used in place of ‘association’ when both considered variables are continuous and that ‘no association’ is equivalent to ‘no correlation’ in the special case of Gaussian variables. If such a confounder variable is not taken into account, an apparent relationship may be observed between the response variable and another predictor variable, but this relationship is in whole or in part the result of the association with the confounder. There are several ways to remove confounding from observed associations between a predictor variable of interest and an outcome, the most popular being adding the confounder as a separate main effect, in addition to the predictor variable of interest, in a multiple regression model with the response variable as a dependent variable.

To better understand the notion of confounding, recall our fetal health example mentioned above. In a naive analysis, a strong positive association could be found between clean hospital floors (predictor variable *X*_3_) and a correct assessment of fetal health (response variable *Y*). However, this spurious association exists due to the fact that trained staff (predictor variable *X*_2_) is associated to both the response variable *Y* and clean hospital floors *X*_3_. This is because, roughly speaking, both *X*_2_ and *X*_3_ depend on the hospital’s quality standards. The predictor variable trained staff thus acts as a confounder. If it is not accounted for, a regression model would show a large regression coefficient for clean hospital floors, whereas if the presence of trained staff is included as an additional predictor variable in the model, we will find that the apparent effect of the clean hospital floors vanishes.

Quite generally and independently of this specific example, the regression coefficient of a predictor variable might be different depending on whether a second predictor variable of interest is included in the model or not. Such a change does not indicate an interaction but is due to the association between these two predictor variables. If they are strongly positively associated and both have, say, a positive effect on the response variable, their coefficients are likely to be smaller in the model including both than in the univariate models including only one of them. This is because in the multiple regression model the regression coefficients correspond to partial effects of one variable given the other(s). This fundamental characteristic of the multiple regression model has also inspired the conditional variable importance measure for RF, which is discussed in section ‘Conditional variable importance measure’.

It is important to note that one is not speaking of a confounder variable if the considered variable is part of the causal pathway. In this case one is speaking of a mediator variable. To explain the term mediator and its role as part of a causal pathway, it is assumed for the moment that the correct assessment of the fetal health shall be predicted from the hospital’s quality standards that could be quantified on a scale. If the hospital’s quality standard is taken as the predictor variable, a well-trained staff is regarded as a mediator because one can expect a hospital with high quality standards to make sure that well-trained staff is employed. As the latter is the decisive factor for a correct diagnosis rather than the hospital’s quality standards, it lies in the causal pathway from the hospital’s quality standards to the correct assessment of fetal health and is thus considered a mediator.

As we have just seen above when discussing the problem of confounding, association/correlation on the one hand and interaction on the other hand, are two completely different concepts: two predictor variables might be independent but show a strong interaction effect and, vice versa, two predictor variables may be strongly associated but have no interaction. To better highlight this issue, let us return to our fetal health example. We have seen that there is a strong interaction between well-trained staff and the availability of high-quality ultrasound devices. It is likely that these two factors are also associated because they are both related to the hospital’s quality standards and because the staff’s competence depends on the opportunity they have had to gain experience with ultrasound devices. In this example we thus have both a strong interaction effect and an association between predictors. But the two concepts of association/correlation and interaction can also occur independently of each other. For example, trained staff and clean hospital floors are associated but do not have any interaction effect: the effect of the staff’s training on the correctness of fetal health assessment does not depend on the cleanliness of the floors and vice versa.

Note that strong association between predictor variables may in some cases hinder the detection of interaction effects, especially in datasets of moderate size. To see this in the case of two binary predictor variables *X*_1_ and *X*_2_, consider a dataset where no observation shows the combination 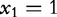 and 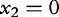. Based on this dataset, it is impossible to determine whether *X*_1_ modifies the effect of *X*_2_, because there is no observation with 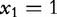 and 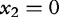, whereas it might be possible with a larger dataset that includes a sufficient number of observations with both 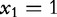 and 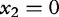. In our example it is unlikely that a hospital buys high-quality ultrasound devices (i.e. 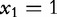) if nobody knows how to use them (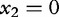). We may thus end up with a dataset where there are no cases with high-quality ultrasound devices but without well-trained staff. These ‘empty cells’—if you imagine a contingency table—make it technically impossible to estimate the interaction effect in generalized linear models and will very likely also affect the results of RF. In these situations, a very large sample size may be necessary to provide a sufficient number of observations with the scarce combination.

### Conditional dependence

One term that is frequently used but not clearly defined in the paper by Touw *et al.* [1] is the term conditional dependence. The way this term is used in the paper does not enable the reader to clearly distinguish its meaning from that of other terms and concepts that are frequently referred to more or less implicitly, such as the concept of interactions or the concept of association/correlation among predictor variables. These concepts, however, are very different, which is relevant for the RF variable importance measures discussed in the paper.

In parts of the statistical literature [10], the term conditional dependence refers to a situation where the association between two variables A and B depends on the value of a third variable C. Here we use the notation A, B and C because we do not yet want to refer to response or predictor variables. At first sight one may directly think of an interaction. But only if either A or B takes the special role as the response variable *Y* then we have indeed an interaction effect as defined in the section ‘Interactions in regression models’ and conditional dependence does become technically equivalent with our definition of interaction. Winham *et al.* [11] take this point of view and use the term conditional dependence to denote interactions. However, if A, B and C are all predictor variables then C only affects the association between two predictor variables and not the association between a predictor variable and a response variable. Thus, in the latter case we cannot speak of an interaction effect in the sense we have outlined in the section ‘Interactions in regression models’.

### Statistical interaction versus biological/genetic interaction

Finally, we should not forget that any statistical finding on the presence of interaction needs to be evaluated for its meaningfulness at a biological or clinical level. For example, in the context of gene–gene interactions (also referred to as epistasis) screening, the challenge is to bridge the gap between statistical interaction and those findings that are relevant from a genetic or biological point of view. Moore [12] indicates the conceptual differences between genetic and biological epistasis on the one hand (both occurring at the individual level and referring to interplays between DNA sequences and/or gene products) and statistical epistasis (occurring at the population level and referring to statistical interactions between DNA-based genetic markers in relation to a response variable of interest) on the other hand. There is no one-to-one correspondence between them.

## TREES AND FORESTS

The aim of the paper by Touw *et al.* [1] was to point out that classification and regression trees and RF offer specific features and require choices about which the user should be well informed. In the remaining sections, we will revisit their key points and show how some are related to the statistical concepts we have described above.

RF is an aggregation of several decision trees. When creating a RF, one can use ‘classical’ trees that use the Gini index as the splitting criterion. Another option is to use conditional inference trees [13]: these are implemented in the R package ‘party’, which also includes the function ‘cforest’, which derives RF from such trees. As we also found potentially misleading statements on conditional inference trees in the paper by Touw *et al.* [1], we briefly review this concept here and clarify the meaning of the word ‘conditional’ used to describe these trees.

### Conditional inference trees

Conditional inference trees are characterized by their particular splitting criterion. They use the *P*-values of a certain type of statistical test as splitting criterion instead of the Gini index as in CART [14]. The specific statistical test depends on the type of response variable (binary, ordinal and nominal categorical, continuous, continuous censored) and on the type of predictor variable (binary, ordinal and nominal categorical, continuous). These tests all fit in a common statistical framework and can be called conditional tests in the sense that the values of the predictor variables and of the response variables are considered as fixed when deriving the null distribution that is used to compute the *P*-value-based splitting criterion. The term ‘conditional’ thus refers to a statistical property of the tests used as splitting criterion for split selection and does neither relate to any type of association/correlation or interaction between variables, nor to the conditional variable importance suggested by Strobl *et al.* [15].

Conditional inference trees were proposed to overcome a serious problem of standard CART and RF algorithms, namely that variables offering more cutpoints are artificially preferred in variable selection. This bias is carried over to the Gini variable importance measure which should therefore not be suggested to applied researchers, although it is still commonly used in practice [16]. In contrast, using conditional inference trees to construct the forests leads to unbiased permutation variable importance measures when used in combination with subsampling instead of bootstrap sampling [17], as is correctly noted by Touw *et al.* [1]. More precisely, the Gini variable importance measure output by the original RF algorithm is strongly biased in favor of predictor variables with many possible splits. For example, in the case of categorical predictor variables, predictor variables with many categories are favored over predictor variables with few categories. But the Gini VIM may also be biased in settings with predictor variables with the same number of categories, for example in SNP data analyses where almost all predictor variables have three categories. In this case, predictor variables with approximately equally sized categories tend to be favored over predictor variables with unequally sized categories [18, 19].

### Conditional variable importance measure

In the presence of associated/correlated predictor variables, another feature of the original RF permutation variable importance measure is that predictor variables that have no effect of their own, but are associated/correlated with an influential predictor variable, can receive a high variable importance. This behavior is not outright wrong, because there are different concepts for judging the importance of a variable in the presence of associations/correlations among the predictor variables (see, for example [20]). However, it is not the behavior a user may expect when he/she is used to the partial or conditional behavior of the regression coefficients in (generalized) linear models that was outlined in the section ‘Interactions in regression models’.

Therefore, Strobl *et al.* [15] proposed an alternative permutation-based variable importance measure called, as we admit potentially misleadingly, ‘conditional variable importance’ that is also implemented in the R package ‘party’.

The term ‘conditional’ here refers to the fact that the variable importance of one variable is computed conditionally on the values of other associated/correlated predictor variables. It was chosen to emphasize the contrast between the partial or conditional view on variable importance on the one hand and the marginal or unconditional view on variable importance on the other hand. The partial or conditional view is inherent in the conditional RF variable importance measure, in partial correlations between one predictor variable and the response variable given another predictor variable or in regression coefficients in multiple regression. In contrast, the marginal or unconditional view is inherent in the standard RF importance measure and in correlations between one predictor variable and the response variable without taking potential confounders into account.

This principle can again be illustrated by recalling the model formula for the logistic regression model with the two predictor variables *X*_2_ (trained staff) and *X*_3_ (clean hospital floors) which do not interact:

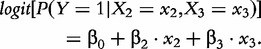

Suppose that 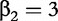 and 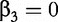, and that *X*_2_ and *X*_3_ are strongly associated. When testing the association between *X*_3_ and *Y* univariately, one would likely find an association—due to the association between *X*_2_ and *X*_3_ on one side and the effect of *X*_2_ on *Y* on the other side, as already outlined in the section ‘Interaction and association/correlation’. However, conditionally on *X*_2_, *X*_3_ does not have any effect on *Y*. Correspondingly, when testing the effect of *X*_3_ in the multiple logistic regression model, one does not expect to find any significance. A multiple regression model assesses the effect of each predictor variable conditionally on the other predictor variables.

The conditional permutation-based VIM proposed by Strobl *et al.* [15] is based on the same principle: it assesses the importance of each predictor variable conditionally on the other predictor variables in order to eliminate the possible influence of association/correlation between predictor variables. The definition of the conditional VIM directly reflects this idea: for each predictor variable that has to be assessed all other predictor variables that are associated are identified and the permutations are performed within groups of observations defined by the values of these predictor variables.

To conclude, the fact that the conditional VIM takes a partial or conditional view on associated/correlated predictor variables has nothing to do with the concept of interactions as we have defined it above.

### Local importance

In the literature on RF, the term local importance refers to the fact that the permutation variable importance suggested for RF by Breiman and Cutler available in the original version of the RF software [21] as well as in the open source implementation [22] cannot only be computed for the entire sample, but also for each observation individually. The importance of each variable then reflects the change in the prediction accuracy for this individual observation averaged over all trees for which the observation was in the out-of-bag-sample. When all individuals from a subgroup of interest are combined, the local importance may indicate that some variables are more important for correctly classifying one subgroup than another.

This idea and its potential for applied research is explained by Touw *et al.* [1]. In their paper, it is motivated by the example of different cancer subtypes for which different predictor variables may be informative. It is important to note that local importance is not directly related to the concepts of association/correlation or interaction in the sense outlined above, but refers to subgroups of the response classes that were not considered in any of the other concepts.

### RF and interaction effects

**Figure 1:**
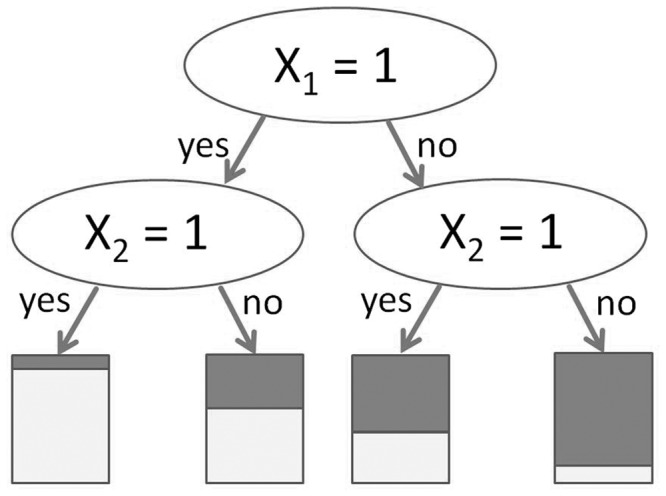
Idealized tree in the presence of two predictor variables, *X*_1_ and *X*_2_ with main effects only (no interaction). The bars at the bottom of the tree denote the proportion of observations with *Y* = 0 and *Y* = 1 in the respective leaves.

**Figure 2:**
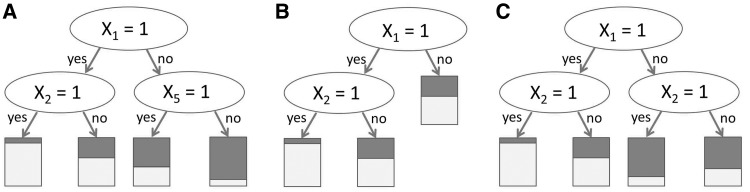
Idealized tree in the presence of two predictor variables, *X*_1_ and *X*_2_ with interaction. (**A**) Different predictor variables are selected on the left and on the right. (**B**) Splitting stops after the first split on the right but not on the left. (**C**) The same predictor variable is selected on the left and on the right, but the effect is different. The bars at the bottom of the tree denote the proportion of observations with *Y* = 0 and *Y* = 1 in the respective leaves.

The split-based structure of classification and regression trees can advantageously take interaction effects into account. Let us consider the first two layers in a tree and how this tree might look when there are only two relevant binary predictor variables *X*_1_ and *X*_2_, with additional irrelevant predictor variables 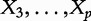. If the root node is split by predictor variable *X*_1_, the effect of *X*_2_ may be different in the two child nodes, hence taking the potential interaction between *X*_1_ and *X*_2_ into account. If *X*_1_ and *X*_2_ have main effects only, one ideally expects *X*_2_ to be selected in both child nodes with the same effect on the response, yielding the idealized picture in Figure 1. Everything else—selection of different predictor variables in the two child nodes, stopping on one side but not on the other, same predictor variable and same cutpoint on both sides but with different effects—indicates a potential interaction (Figures 2A, B and C as examples of these three situations) [23]. The problem is that, due to random variations in finite samples, it is extremely rare that the tree selects the same predictor variable with the same effect on both sides, except perhaps in the case of very large samples. Moreover, the fact that in RF the splitting variable is selected out of only *mtry* candidate variables—that are randomly selected for each split—increases the differences between the branches of a tree: If *mtry* is set smaller than the total number of predictor variables, we are sure that the ideal pattern of Figure 1 will not always be observed even for infinite sample sizes, because *X*_2_ will not always be in the subset of candidate predictor variables for the splits of the second layer. Thus, in practice a tree almost always looks as if there were interactions as it includes patterns as in Figure 2, but empirically such patterns will also be seen in the absence of interactions. The essential question is thus whether these patterns are just the result of random variations (chance) and of the recursive nature of the tree building algorithms, or of true interactions. This question is far from trivial and to date there exists no standard approach to answering it only by investigation of the trees of a RF.

RF methodologies are commonly claimed, often in rather vague terms, to be able to handle interactions [24–29], although, by construction, the predictor defining the first split of a tree is selected as the one with the strongest main effect on the response variable [30]. It is not within the scope of this letter to answer whether this claim is justified or not. However, we feel that whenever RF methodologies are investigated in relation to interactions, the latter term should be defined precisely and the investigated role of RF in this context should be clearly stated. For example, does it relate to the ability of RF to yield high individual VIMs for predictor variables involved in interactions [31], the possibility to directly identify which predictor variables interact with each other by examining a RF [32, 1], or the combination of RF with other analysis tools with the aim of identifying interactions [30]? In any case, when an algorithm based on RF (possibly combined with other tools) is suggested to identify which predictor variables interact with each other, we claim that this algorithm should be assessed in simulations using adequate measures such as, for example, sensitivity, the proportion of pairs of interacting variables that are correctly identified as interacting; specificity, the proportion of pairs of non-interacting variables that are correctly identified as non-interacting; or false positive rate, the proportion of pairs of non-interacting variables within the pairs identified as interacting.

## CONCLUSION

Clearly, regarding interactions and associations between variables, the terminology found in the literature is highly heterogeneous and is best carefully specified as a preliminary to all further considerations, while keeping in mind that each community (bioinformaticians, statisticians, machine learners, geneticists, epidemiologists, etc.) may understand apparently unequivocal terms in different ways. To prevent misunderstandings, we appeal to researchers in this area to clearly define what they mean by any kind of statistical terms and avoid using ambiguous and imprecise phrasings. Through this work we hope to have clarified the most central statistical concepts that are necessary for understanding issues related to interactions and RF.

**Key Points**

- Concepts such as interaction or conditional dependence have ambiguous meanings. A careful definition of these concepts as a preliminary to all further considerations in an article might avoid misunderstandings.
- Different definitions of these terms are conceivable, but within an article definitions should be consistent.
- The word ‘conditional’ has different meanings in the term ‘conditional inference trees’ and in the term ‘conditional variable importance measure’. They should not be confused.
- The extraction of information on interactions between predictor variables based on random forests is not trivial.

## FUNDING

S.J. was supported by grant BO3139/2-2 from the German Science Foundation to A.L.B. and by Biomed-S.
